# Single-cell and bulk RNA sequencing analysis of B cell marker genes in TNBC TME landscape and immunotherapy

**DOI:** 10.3389/fimmu.2023.1245514

**Published:** 2023-12-04

**Authors:** Fangrui Zhao, Chen Zhao, Tangpeng Xu, Yanfang Lan, Huiqing Lin, Xiaofei Wu, Xiangpan Li

**Affiliations:** ^1^Department of Oncology, Renmin Hospital of Wuhan University, Wuhan, Hubei, China; ^2^Department of Thoracic Surgery, Renmin Hospital of Wuhan University, Wuhan, China; ^3^Department of Neurology, Central War Zone General Hospital of the Chinese People's Liberation Army, Wuhan, Hubei, China

**Keywords:** B cell marker genes, TNBC, single-cell sequencing, cancer immunotherapy, predictive score

## Abstract

**Objective:**

This study amied to investigate the prognostic characteristics of triple negative breast cancer (TNBC) patients by analyzing B cell marker genes based on single-cell and bulk RNA sequencing.

**Methods:**

Utilizing single-cell sequencing data from TNBC patients, we examined tumor-associated B cell marker genes. Transcriptomic data from The Cancer Genome Atlas (TCGA) database were used as the foundation for predictive modeling. Independent validation set was conducted using the GSE58812 dataset. Immune cell infiltration into the tumor was assessed through various, including XCELL, TIMER, QUANTISEQ, CIBERSORT, CIBERSORT-ABS, and ssGSEA. The TIDE score was utilized to predict immunotherapy outcomes. Additional investigations were conducted on the immune checkpoint blockade gene, tumor mutational load, and the GSEA enrichment analysis.

**Results:**

Our analysis encompassed 22,106 cells and 20,556 genes in cancerous tissue samples from four TNBC patients, resulting in the identification of 116 B cell marker genes. A B cell marker gene score (BCMG score) involving nine B cell marker genes (*ZBP1, SEL1L3, CCND2, TNFRSF13C, HSPA6, PLPP5, CXCR4, GZMB*, and *CCDC50*) was developed using TCGA transcriptomic data, revealing statistically significant differences in survival analysis (*P*<0.05). Functional analysis demonstrated that marker genes were predominantly associated with immune-related pathways. Notably, substantial differences between the higher and lower- BCMG score groups were observed in terms of immune cell infiltration, immune cell activity, tumor mutational burden, TIDE score, and the expression of immune checkpoint blockade genes.

**Conclusion:**

This study has established a robust model based on B-cell marker genes in TNBC, which holds significant potential for predicting prognosis and response to immunotherapy in TNBC patients.

## Introduction

Triple-negative breast cancer (TNBC) is a distinct subtype of breast cancer characterized by the absence of estrogen receptor (ER), progesterone receptor (PR) and human epidermal growth factor receptor 2 (HER2) expression in immunohistochemistry (1). Accounting for approximately 15–20% of all breast cancers, TNBC exhibits aggressive clinical symptoms (2). Although chemotherapy remains a major therapeutic method for metastatic TNBC; its efficacy is limited, yielding a median overall survival time of 12-18 months (2). Consequently, there is an urgent need for innovative therapeutic strategies.

In recent years, immunotherapy has emerged as a promising intervention, demonstrating prolonged survival in various solid tumors (3–5). Notably, immune checkpoint inhibitor (ICI) monotherapy in TNBC patients has shown response rates ranging from 5% to 23% (6), while combined with chemotherapy, for early-stage TNBC patients exhibit pathologically complete response rates between 22% and 60% (7). Despite these advances, only a subset of patients benefits from immunotherapy, highlighting the need for the development of predictive models and the identification of novel biomarkers to better anticipate treatment outcomes and prognosis.

Single-cell RNA sequencing (scRNA-seq) technology and related data analysis methods has facilitated the exploration of molecular characteristics of immune cells within the tumor microenvironment (TME) (8). This unparalleled opportunity has provided insights into cancer immunity, enabling the establishment of genetic markers based on immune cell molecular characteristics to predict immunotherapy outcomes in cancer patients (9, 10).

Recent research has demonstrated that tumor-infiltrating B lymphocytes (TIL-Bs) in breast cancer, responding to B cell receptor (BCR) activation and generating immunoglobulin (Ig) *in vivo* (11–13). Thus, B cells may significantly influence the prognosis of breast cancer, particularly in patients with immunogenic TNBC. Here, our study aimed to conduct an integrated analysis of scRNA-seq data from TNBC samples, identify B cell marker genes, and subsequently develop a predictive model for accessing the prognosis of TNBC patients.

## Methods

### Data collection

Transcriptional RNA sequencing data from single-cell profiling datasets of four TNBC tumor tissue (GSM4909281, GSM4909282, GSM4909283 and GSM4909284; accessible at https://www.ncbi.nlm.nih.gov/geo/) were acquired for the purpose of investigating B cell marker genes. Concurrently, clinical profiles and transcriptomic data were obtained from the Cancer Genome Atlas (TCGA) and Gene Expression Omnibus (GEO) databases.

### Processing single-cell sequencing data

The single-cell sequencing data obtained from patients were subjected to comprehensive analysis through the R software and related packages. Seurat objects were constructed following the parsing of single-cell sequencing data sourced from TNBC samples represented by GSM4909281, GSM4909282, GSM4909283, and GSM4909284. The DoubletFinder package was applied to eliminate the cell doublets within the sample. Exclusion criteria for cells of suboptimal quality are as follows: 1) the number of features exceeding 500 and falling below 6000, 2) the mitochondrial genes expression below 10%, 3) the expression of erythroid genes less than 5%. In order to integrate data from multiple samples, the software known as “harmony” was employed.

The Uniform Manifold Approximation and Projection (UMAP) method was adopted to reduce the number of dimensions, displaying clustered cells with only two dimensions on a map. The subpopulations of tumor-associated B cells were determined by using the “SingleR” software as previous publications indicated (14, 15). Marker genes for different cell types were ascertained through Wilcoxon-Mann-Whitney test and the “FindAllMarkers” function analysis. The criteria for filtering marker genes of various cell types included a | log2 (fold change) | > 1 and the adjusted P value less than 0.05.

### Functional enrichment

The functional annotation of marker genes associated with tumor-associated B cells was augmented through enrichment analysis using the Gene Ontology (GO) and the Kyoto Encyclopedia of Genes and Genomes (KEGG). The c2.cp.v2023.2.Hs.symbols.gmt dataset from the MSigDB database (https://www.gsea-msigdb.org/gsea/msigdb) was used for Gene Set Enrichment Analysis (GSEA) to elucidate the biological processes involved in the B-cell marker gene.

### A predictive model of B cell marker gene

A predictive model for B cell marker gene expression in TNBC was developed to predict TNBC prognosis. In the univariate COX regression analysis, genes identified as B-cell markers were incorporated. The least absolute shrinkage and selection operator (LASSO) regression was performed to construct a penalty function, effectively compressing the coefficients of variables and reducing overfitting of the model caused by prognosis-related genes. Subsequently, the outcomes from the multivariate COX regression analysis were used to formulate a predictive B cell marker gene score (BCMG score) model.

The formula is as follows:


$$BCMG\;score=\beta_{1}*\;Expr_{1}+\beta_{2}*\;Expr_{2}+\ldots +\beta_{n}*Expr_{n}$$

Here, *Expr* denotes the mRNA expression of the crucial gene, *n* denotes the number of genes included in the model, and *β* represents the associated regression coefficient determined in the multivariate gene COX regression analysis. The data was then classified into higher and lower- BCMG score groups based on the median value of the BCMG score.

To validate the model’s robustness, an independent third-party validation set, GSE58812, was employed to assess and confirm the predictive performance of the model.

### Immune infiltration analysis

Based on the gene expression dataset of patients, the CIBERSORT algorithm was conducted to determine the magnitude of immune cell proliferation in each sample. Spearman correlation analysis was performed to examine the variations in immune cell infiltration between the two groups. A single-sample GSEA was carried out to further assess the differences in immune cell activity between the higher and lower- BCMG score groups. Estimation techniques were implemented to determine the differences of immune scores, stromal scores, overall scores, and tumor purity between the two groups. Multiple tools, including XCELL, TIMER, MCP counter, CIBERSORT, and CIBERSORT-abs, were applied to establish a correlation between B cell marker genes and immune cells.

### Forecasting the patient’s response to immunotherapy

Spearman test was used to analyze the correlation of expression between model and immune genes. The TIDE score was incorporated to evaluate the effectiveness of immunotherapy in individual patients. Patients with lower TIDE scores indicated a decreased risk of immunological escape, which suggested a higher likelihood of effective immunotherapy. An online tool TIDE (http://tide.dfci.harvard.edu/) was used to examine the immunotherapy scores of each patient, and investigate the differences in immunotherapy results between the higher and lower- BCMG score groups.

### Analysis of the tumor’s mutational burden

The raw data of the patient’s tumor mutations were obtained and downloaded from the TCGA database. The “maftools” package in R was used to generate waterfall graphs in accordance with predefined specifications. This study also further assessed the differences in tumor mutation burden as well as predicted discrepancies between higher and lower- BCMG score groups.

### Statistics analyses

Kaplan-Meier method and the log-rank test were utilized for survival analysis. Alternately, the Wilcoxon rank-sum test was employed to compare the differences existing between the two groups. R (4.1.2, available at https://www.r-project.org/) was conducted throughout the entire data analysis process. P values of less than 0.05 on both sides were defined as statistically significant.

## Results

### Examination of single cells

**Figure 1:**
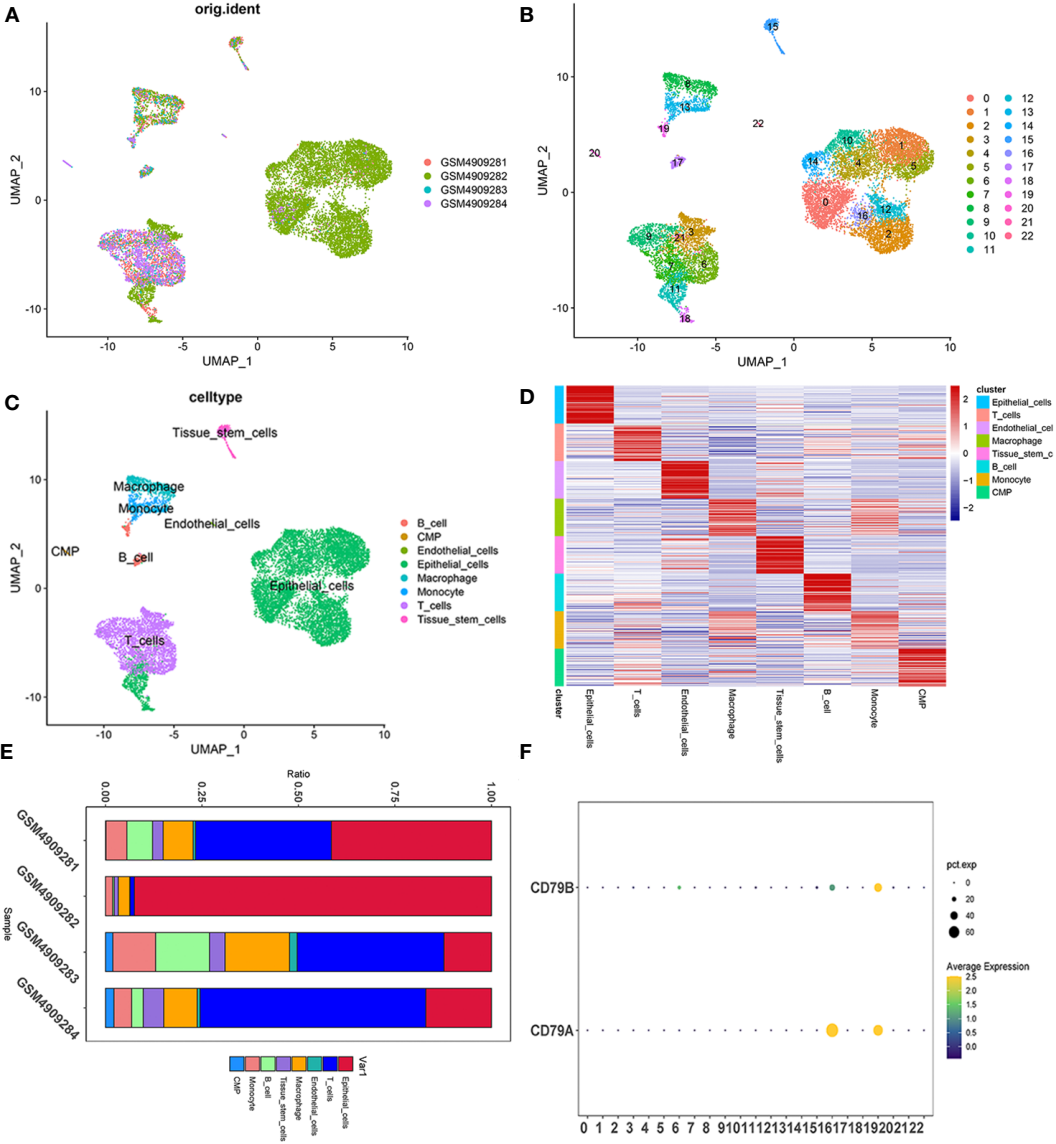
Single cell sequencing profile of 22,106 cells from human samples of TNBC. **(A–C)** UMAP plots of 22,106 tumor-associated B cells here, colored by individual samples **(A)**, 22 cell clusters identified when resolution equals 2 **(B)** and cell types **(C)**. **(D)** Heatmap displaying the top 10 marker genes in each cell cluster. **(E)** Proportions of each cell type in each sample colored by cell types. **(F)** Expression analysis of BCMGs in 22 cell clusters. The intensity of the color indicates the average expression of the gene. Dot size indicates the percentage of cells expressing the gene.

The integration of single-cell sequencing data from four TNBC patients was achieved using the “Harmony” package. After excluding cells of suboptimal quality, the subsequent study consisted of the examination of 22,106 cells and 20,556 genes (**Figure 1**). The identification of potential marker genes for TNBC-associated B cells was conducted through the application of the Wilcoxon-Mann-Whitney test, revealing 116 distinct genes with significant differences (**Table S1**). The clinical characteristics of the training group TCGA and the testing group GSE58812 was detailed in **Table S2**.

### Tumor-associated B-cell marker gene enrichment analysis

**Figure 2:**
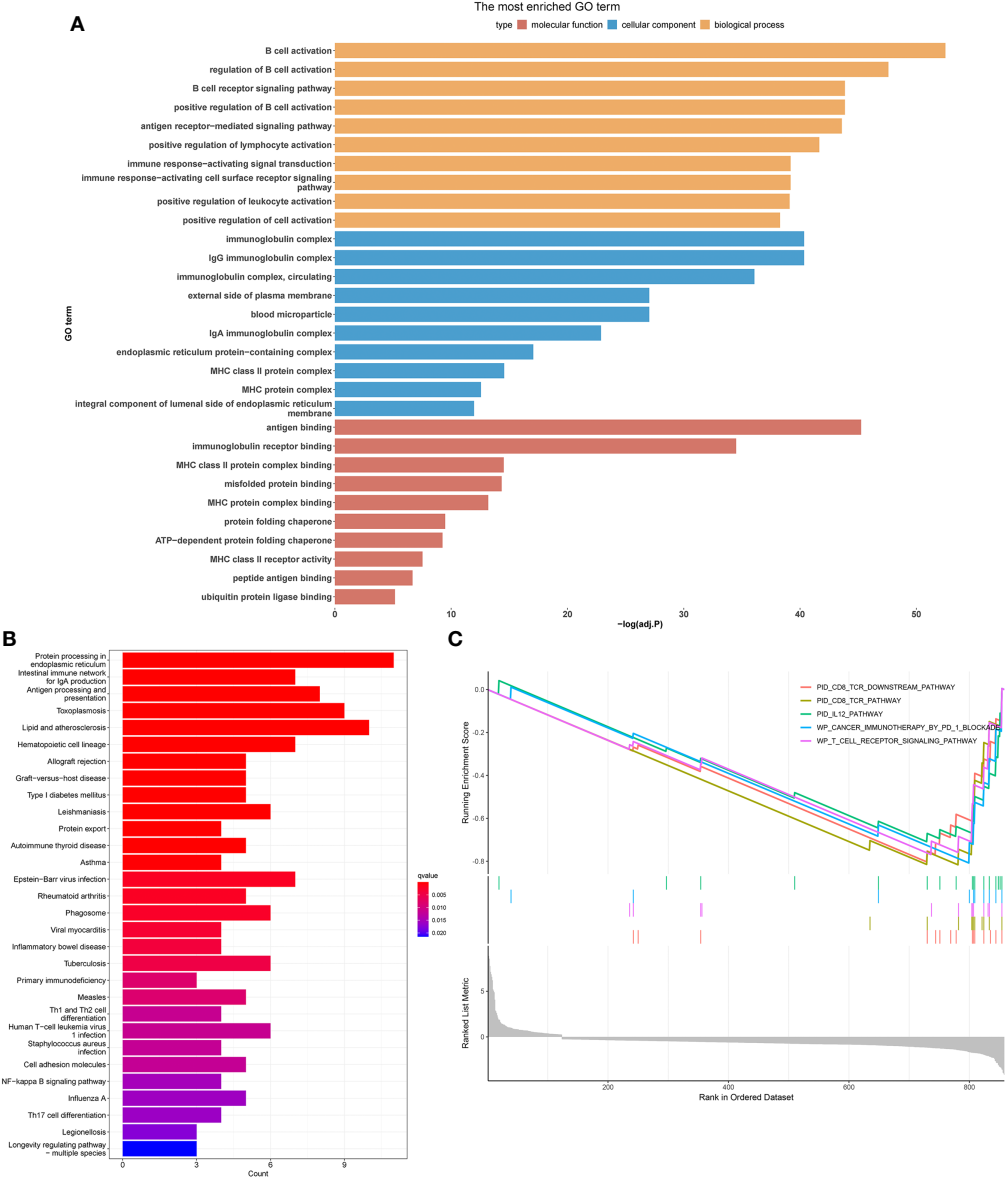
Exploring the differences in activity in different cell types by employing different enrichment analysis methods. Histogram of GO **(A)**, KEGG **(B)** and GSEA **(C)** enrichment analysis.

The investigation of genes enriched in tumors associated B cells yielded notable results. The GO and KEGG analysis revealed that marker genes of B cells predominantly influenced functional pathways specific to B cells. Significant differences were observed in cell pathway activity scoring across different cell types. Particularly, the unfolded protein response signaling pathway demonstrated notable activity in B cells. The B cell marker genes mainly participate in biological processes such as pid CD8 tcr dowstream pathway, pid IL12 pathway, WP cancer imunotherapy by PD 1 blockade, and WP T cell recpto signaling pathway. (**Figure 2**).

### Development of a predictive model for B-Cell marker gene expression

**Figure 3:**
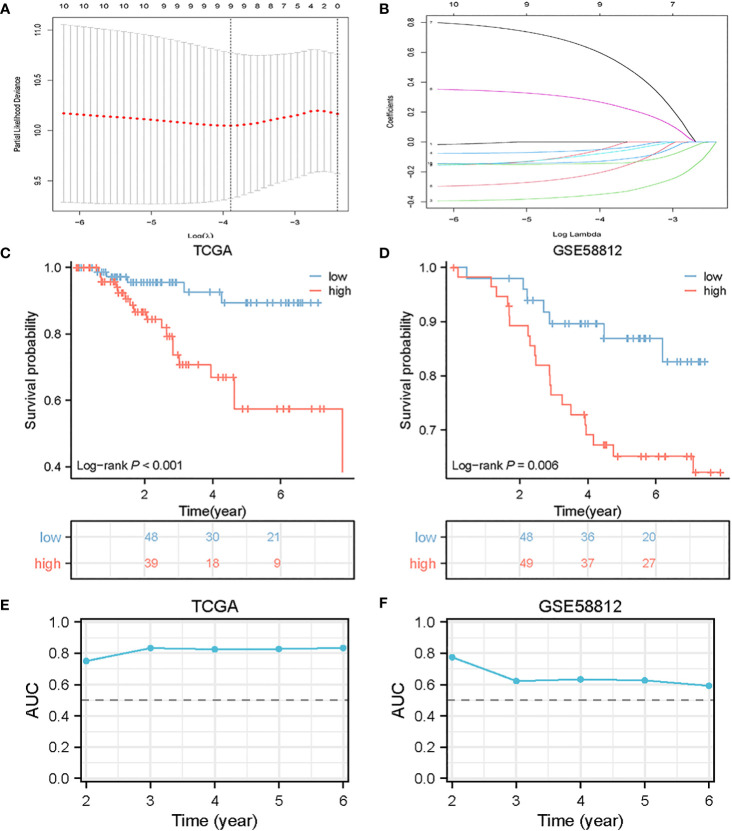
Construction of BCMG score model in the TNBC cohort. **(A)** Log (λ) change curves of regression coefficients and cross-validation for optimizing the parameter in LASSO regression. **(B)** Tenfold cross-validation of adjusted parameter choices in lasso regression. **(C, D)** Kaplan-Meier survival analysis of TCGA and GSE58812 of TNBC patients stratified by higher and lower- BCMG score. **(E, F)** ROC curve analysis for predicting the risk of death in the cohort of TCGA **(E)** and GSE58812 **(F)**.

The evaluation of the 116 genes involved a univariate COX regression to identify the 10 genes associated with prognosis. In order to prevent overfitting, a lasso regression analysis was subsequently performed. The results of the lasso regression were further subjected to a multifactor COX regression analysis to construct predictive models. The final model incorporated the following variables: *ZBP1, SEL1L3, CCND2, TNFRSF13C, HSPA6, PLPP5, CXCR4, GZMB*, and *CCDC50* (**Figures 3A, B**).

The predictive score was computed based on the expression levels of the selected genes using the following formula:


$$\begin{array}{c} \text{BCMG score}=(-0.0390\times ZBP1)+(-0.3450\times SEL1L3)+(-0.0474\times CCND2)+(-\\ 0.0716\times TNFRSF13C)+(0.2560\times HSPA6)+(0.5725\times PLPP5)+(-0.1924\times \\ CXCR4)+(-0.1424\times GZMB)+(-0.1246\times CCDC50) \end{array}$$

Patients from TCGA and GEO were divided into higher and lower- BCMG score groups according to the median value of the scores obtained from TCGA.

### Model validation

**Figure 4:**
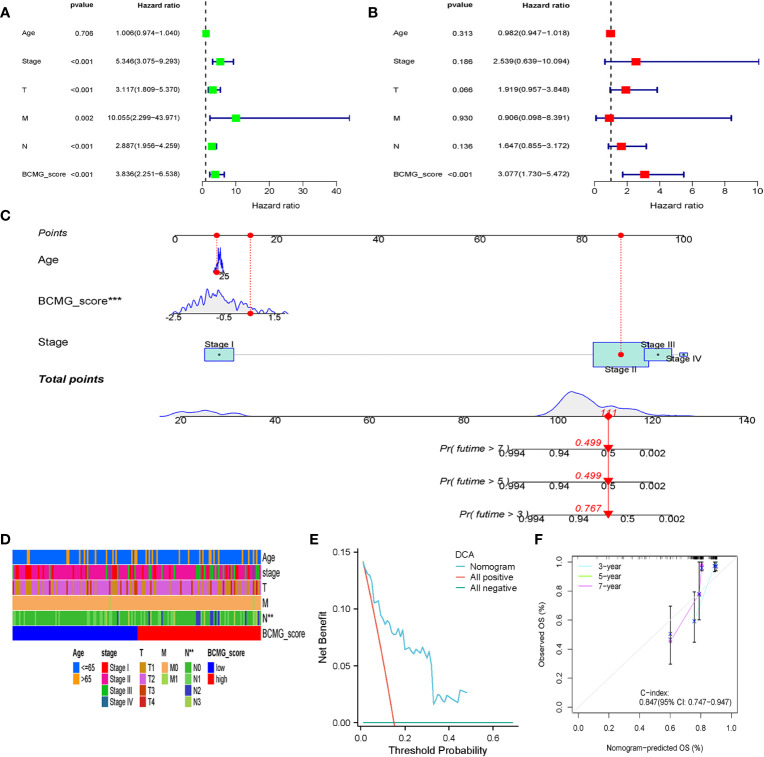
Clinical analysis in the cohort of TCGA. **(A)** Univariate Cox regression analysis revealed the association between patients’ survival and clinicopathological parameters along with BCMG score. **(B)** Multivariate Cox regression analysis uncovered that only the BCMG score (P< 0.001) was an independent prognostic factor for TNBC patients. **(C)** The prediction of 3-, 5-, and 7-year survival for TNBC patients based on the prognostic nomogram derived from the BCMG score and other clinicopathologic feature. **(D)** Clinical correlation with age, stage, T, N, M and BCMG score shown in heatmap. **(E)** DCA curve analysis. **(F)** Calibration Curve illustrated the consistency between predicted and observed 3-, 5-, and 7- year survival rates in TNBC patients depending on the prognostic nomogram.

**Figure 5:**
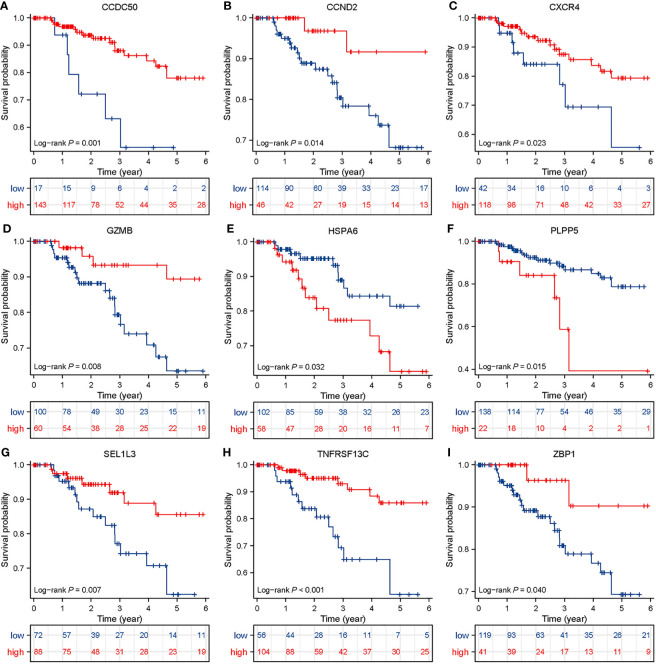
Survival analysis in TNBC patients based on BCMG score. Kaplan-Meier survival analysis of TCGA TNBC patients stratified by high and low *CCDC50, CCND2, CXCR4, GZMB, HSPA6, PLPP5, SEL1L3, TNFRSF 13C*, and *ZBP1* **(A–I)**, which were based on the best cutoff values.

**Figure 6:**
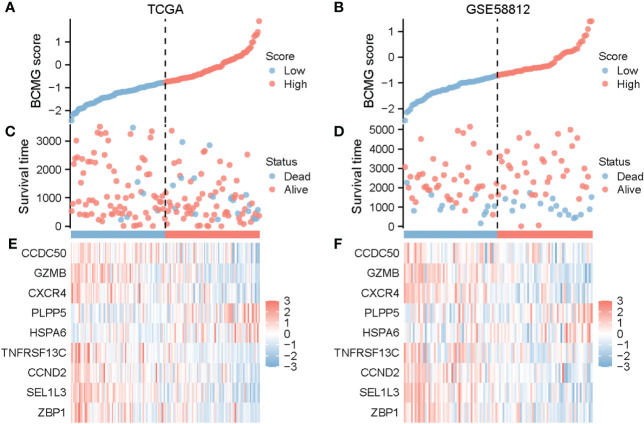
Prognostic analysis of TNBC patients on the basis of BCMG score in TCGA and GEO. BCMG score distribution in TCGA **(A)** and GSE58812 **(B)**. Scatterplot of survival status and survival time of TNBC patients in TCGA **(C)** and GSE58812 **(D)**. Heatmap generated on the basis of identified gene expression in TCGA **(E)** and GSE58812 **(F)**.

The survival curves revealed that patients classified in the higher BCMG score group had a worse prognosis than those in the lower BCMG score group (**Figures 3C, D**). The time-dependent receiver operating characteristic (ROC) curves indicated that the predictive model had an outstanding discriminatory capability (**Figures 3E, F**). Importantly, these results were not only internally validated within the study group but also externally validated in an independent validation group (**Figures 3E, F**). According to the findings of the independent predictive study (**Figures 4A, B**), the predictive model was identified as a significant independent predictive factor in patients with TNBC. The correlation analysis with clinical characteristics further highlighted the close association between the predictive model and lymph node metastases (**Figure 4**). In-depth exploration of the correlation between the model genes and patient prognosis (**Figure 5**) revealed that the expression levels of *ZBP1*, *SEL1L3*, *CCND2*, *TNFRSF13C*, *CXCR4*, *GZMB*, and *CCDC50* were higher in the lower BCMG score group compared to the higher group. Conversely, the higher BCMG score group exhibited significantly higher levels of expression for *HSPA6* and *PLPP5* (**Figure 6**).

### Analysis of tumor mutation burden

**Figure 7:**
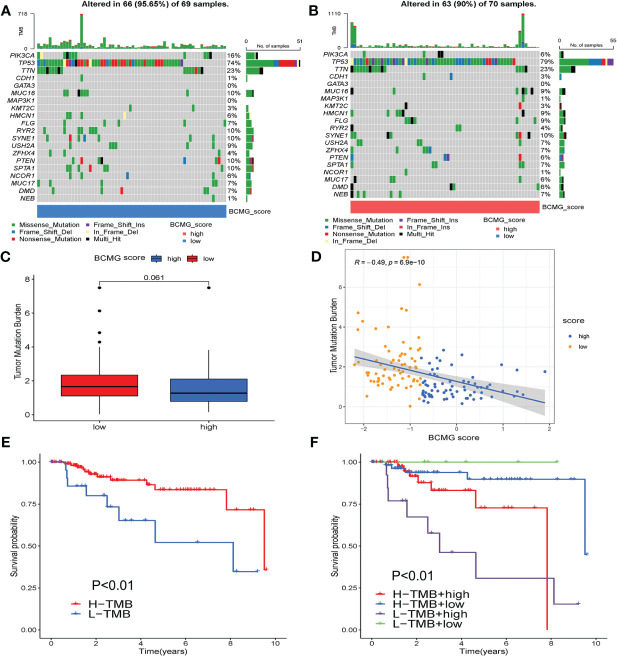
Tumor mutation burden analysis. **(A, B)** Differential counting of tumor mutation burden between higher and lower - BCMG score groups. **(C)** Tumor mutation load comparison. **(D)** Correlation analysis of BCMG score and mutation burden. **(E, F)** Prognostic analysis of tumor mutation load in higher and lower- BCMG score groups.

The analysis of the tumor mutation burden revealed an inverse relationship with the prognostic model. The waterfall plot visually illustrated that the mutation types were not identical between the higher and lower- BCMG score groups. The survival analysis results indicated that patients with lower tumor mutation load exhibited a more favorable prognosis (**Figure 7**).

### Analysis of TME

**Figure 8:**
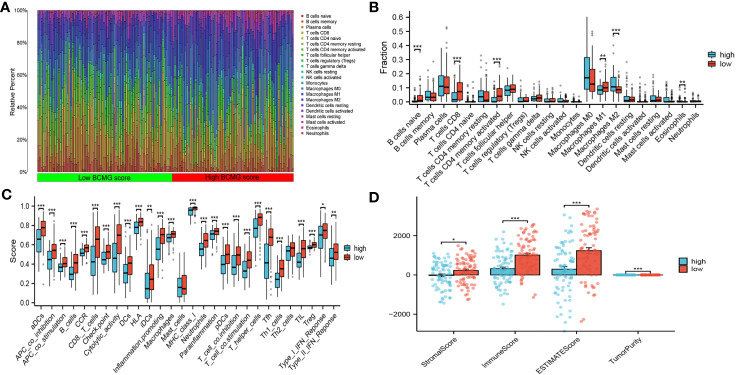
Correlation of the B cell prognostic model genes with immune cell infiltration and immune checkpoint in TCGA cohort. **(A)** Analysis of immune cell infiltration in higher and lower- BCMG score groups. **(B)** The comparison of 22 immune cells’ infiltration level in higher and lower- BCMG score groups. **(C)** The comparison of 28 immune cells’ infiltration level in higher and lower- BCMG score groups. **(D)** TME score differences compared based on the ESTIMATE algorithm. *P < 0.05; **P < 0.01; ***P < 0.001.

**Figure 9:**
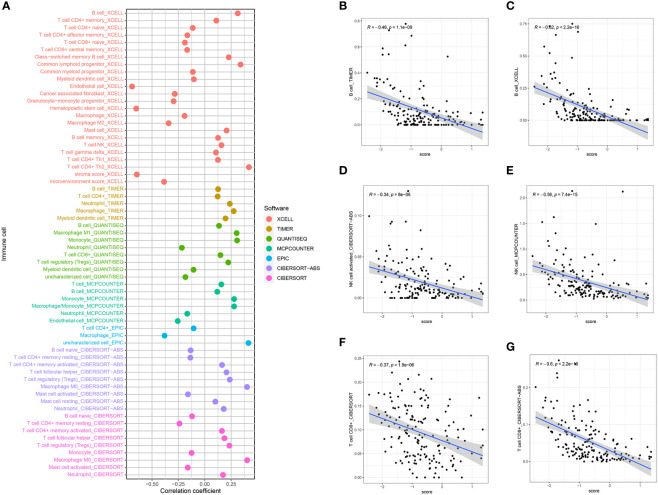
BCMG score and immune cell correlation analysis. **(A)** Spearman correlation analysis showed that BCMG scores strongly correlated with tumor-infiltrating immune cells. **(B–G)** Correlation of predictive models and immune cells based on XCELL, TIMER, QUANTISEQ, CIBERSORT, and CIBERSORT-abs.

The CIBERSORT analysis demonstrated the differential presence and activity of various immune cell types in the TMB between the higher and lower- BCMG score groups. Specifically, CD8^+^ T cells, activated memory CD4^+^ T cells, M1 macrophages, M2 macrophages and eosinophils were found to serve different functions between the two groups (**Figures 8A, B**). The overall activity of the majority of immune cells exhibited notable changes in both groups, further emphasizing the dynamic nature of the TME (**Figure 8C**). The ESTIMATE algorithm indicated that the lower BCMG score group had significantly higher tumor scores, interstitial scores, total scores, and tumor purity scores than the higher BCMG score group (**Figure 8D**). Furthermore, correlation analysis between the predictive scores and the TME analyzed by the four methods CIBERSORT-ABS, CIBERSORT, QUANTISEQ, and XCELL, demonstrated the potential influence of B-cell marker gene model on the TME (**Figure 9**).

### Predictions regarding immunotherapy

**Figure 10:**
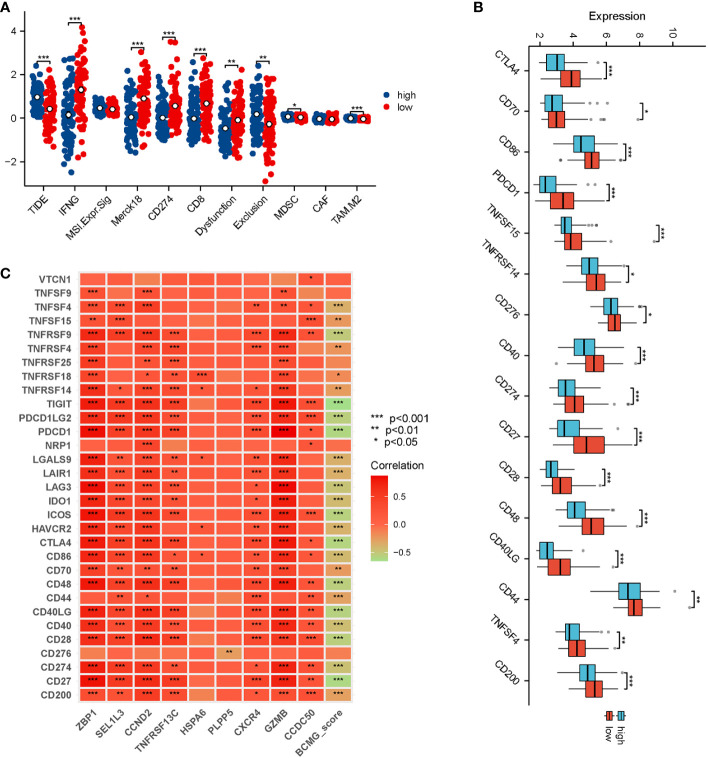
Prediction of response of TNBC patients to immunotherapy. **(A)** Analysis of differences in TIDE, IFNG, MSI Expr, Merck18, CD274, CD8, Dysfunction, Exclusion, MDSC, CAF, and TAM M2 between higher and lower- BCMG score groups. **(B)** Analysis of immune checkpoint blocking gene expression between higher and lower- BCMG score groups. **(C)** Relevance of model genes and immune checkpoint blocking gene.

Correlation analysis revealed that *ZBP1, GZMB, CCND2*, and *TNFRSF13C* exhibited strong correlations with the majority of the genes that inhibit immune response (**Figure 10A**). Simultaneously, a significant difference was observed between the higher and lower- BCMG score groups concerning the expression of several immune blockade checkpoint genes (**Figure 10C**). The TIDE ratings were used to make predictions about the outcomes of immunotherapy treatment for patients classified as either high or low BCMG expression. The TIDE scores of patients in the high expressed group were significantly higher than those of patients in the low expressed group, which indicated a higher likelihood of immune evasion in the high expressed group and immunotherapy may be less effective for patients at high expression. In addition, there were observed differences between the two groups in terms of CD8 scores, CD274 ratings, MDSC scores, T cell dysfunction scores, and T cell exclusion scores (**Figure 10B**).

## Discussion

In this study, we investigated the role of B-cell marker genes in TNBC using scRNA-seq technology. We successfully established a novel prediction score for TNBC patients, derived from the identified B-cell marker genes within the TCGA database. This prediction score was rigorously validated for its predictive capabilities in an independent cohort of the GEO dataset. Our analysis highlighted specific genes, including *ZBP1, SEL1L3, CCND2, TNFRSF13C, CXCR4, GZMB*, and *CCDC50*, which exhibited protective characteristics. In contrast, *HSPA6* and *PLPP5* were identified as having negative implications. Subsequently, the nine gene signatures collectively contribute to a robust predictive model that emerges as a promising predictor for clinical prognosis in TNBC patients.

Patients with TNBC exhibit a significantly higher level of immune infiltration compared to other breast cancer subtypes, despite their overall poor prognosis. The higher immune infiltration is characterized by elevated tumor-infiltrating lymphocytes (TILs) (16), increased levels of PD-L1 expression in tumor cells and immune cells (17, 18), and a greater number of non-synonymous mutations, which produce tumor-specific neoantigens, activate neoantigen-specific T cells, and trigger anti-tumor immune responses (19, 20). This unique immune landscape suggests a greater potential for benefit from immunotherapy in TNBC comparison to other subtypes. ICIs can block immunosuppressive receptors and increase the cellular toxicity and proliferation of TILs (3, 21, 22). According to the findings of a few studies, the effectiveness of ICI monotherapy ranges from 5% to 23% (6), whereas the effectiveness of combination therapy ranges from 22% to 60% (7). Nonetheless, some patients experienced resistance during or after treatment, leading to immune escape and tumor recurrence. Dual checkpoint blockade, which have synergistic anti-tumor effects in advanced malignancies, such as anti-CTLA-4 and anti-PD-1, could potentially serve as a more effective therapeutic strategy (23). Furthermore, recent studies have explored innovative strategies, such as bispecific antibodies targeting TGF-β and PD-L1 (BiTP), which have demonstrated potent antitumor activity in TNBC. BiTP in murine TNBC models showed higher anti-tumor activity compared with solo anti-PD-L1 or anti-TGF-β (24). The rejection and exhaustion of CD8^+^ T cells are two key factors that contribute to the reduced tolerance of ICI therapy. When enhanced CD8^+^ T-cell infiltration occurs, there is a shift towards heat immunity (25). BiTP has a high binding affinity to the dual targets, which reduces collagen deposition, enhances CD8^+^ T-cell penetration and increases TILs (26).

Our study provides valuable insights into the molecular landscape of TNBC-associated B cells and their functional implications in tumor biology. These findings underscore the intricate interplay between the identified prognostic model and the immune landscape within the TME, and we also illustrate potential immunomodulatory roles of the B-cell marker genes in the context of TNBC. Moreover, the differential mutation burden observed in distinct BCMG score groups highlights the importance of considering mutational characteristics in conjunction with the prognostic model for a comprehensive understanding of the disease prognosis in TNBC patients.

Extensive investigations in the breast cancer TME have primarily focused on CD8^+^ T lymphocytes and natural killer (NK) cells (27). In contrast, TIL-B lymphocytes (TIL-Bs) cells may exhibit antigen-induced phenotype (28), and it is hypothesized that autoantibodies play a role in initiating tumor cell clearance (29). TIL-Bs have also been identified as antigen-presenting cells, contributing to the stimulation of an immune response against tumors (30). Moreover, there is a potential for TIL-Bs to form stromal clusters with T cells, engaging in functional cross-talk in both directions. The formation of BCR-immune complexes may also be facilitated by TIL-Bs through the upregulation of BCR pathway components (1). Besides, CD8^+^ T cells represent pivotal defense cells in TME and establish a functional cycle with dendritic cells (DCs) and NKs (31). Positive crosstalk is observed between CD8^+^ T cells and DCs, with CD8^+^ T cells inducing NK cell activity (32). Therefore, it is possible that B cells exert a significant influence on the prognosis of breast cancer, particularly in patients of immunogenic TNBC.

The scRNA-seq technology has allowed for a more in-depth exploration of the molecular properties of immune cells within the TME, offering potential insights into novel biomarkers (33). In this study, the investigation of B-cell marker genes provides valuable insights into the potential role of nine specific genes in influencing the prognosis of individuals diagnosed with TNBC. *ZBP1*, overexpressed in necrotic breast tumors, has been linked to the induction of interferon. In the absence of *ZBP1*, both the process of tumor necrosis and the suppression of metastasis are prevented (34). *SEL1L3*, with decreased expression in cancer cells during sustained endoplasmic reticulum stress (35), may serve as a predictive tool for survival outcomes and immunotherapy response in various cancers (36–39). The promoter of *CCND2* is hypermethylated (40–42) in solid tumors, which leads to *CCND2* hypo-expression (40, 43, 44) and promotes cell proliferation (45). *TNFRSF13C* could encode B-cell activating factor receptor, which regulates B-cell proliferation, development and maturation (46, 47). High levels of *CXCR4* levels were expressed in more than 40% of breast tumor tissues (48) and in 75% of TNBC patients (49), and overexpression of *CXCR4* in cancer cells contributes to tumor growth, invasion, metastasis, and recurrence (50). *GZMB*, known for stimulating anti-tumor immune responses and inhibit tumor growth (51), is correlated with favorable prognosis in breast cancer tissues (52, 53). *CCDC50* is known to mediate apoptosis via the NF-κB pathway (54) and had prognostic predictive value in lung adenocarcinoma (55). The two-way role of *HSPA6* in tumor, acting as both a potential target for tumor inhibition and risk factor of tumor development and tumor progression, underscores the complexity of its functions in cancer biology (56). Our study suggested a negative correlation between high *HSPA6* expression and prognosis, which contradicts the findings reported by Shen et al., where high *HSPA6* expression was positively correlated with longer overall survival (57). This discrepancy may arise from the multifaceted functions of *HSPA6*, affecting different aspects of cancer biology. *PLPP5* participates in the regulation of many cancer-associated transduction pathways such as the JAK/STAT (58), and its overexpression in pancreatic and small cell lung cancer cells may promote proliferation and survival (59). However, in breast cancer cell lines, down-regulation of *PLPP5* inhibits tumor growth and increases apoptosis (60), which is consistent with our study.

Despite these intriguing findings, there are certain limitations in the current study. Additional research is needed to get a deeper understanding of molecular interactions and mechanisms underlying these gene functions. Furthermore, it is essential to verify these findings in cell lines, animal models, and tissue samples to ensure their relevance and applicability to clinical settings.

## Conclusion

In conclusion, this study established a robust model utilizing B cell marker genes to predict prognosis and immunotherapy response in TNBC patients, contributing novel insights into the understanding of TNBC molecular landscape and its implications for therapeutic interventions. The discovery of these gene signatures adds to our understanding of TNBC and offers a promising avenue for improving patient outcomes and guiding therapeutic strategies in this challenging subtype of breast cancer. Further research and clinical validation may continue to refine the application of these gene signatures in the clinical management of TNBC patients.

## Data availability statement

The datasets presented in this study can be found in online repositories. The names of the repository/repositories and accession number(s) can be found in the article/**Supplementary Material**.

## Ethics statement

Ethical approval was not required for the study involving humans in accordance with the local legislation and institutional requirements. Written informed consent to participate in this study was not required from the participants or the participants’ legal guardians/next of kin in accordance with the national legislation and the institutional requirements.

## Author contributions

Conceptualization: CZ and HL; Data curation: TX; Methodology: XW; Formal analysis: YL; Writing-original draft: FZ; Review and editing of the manuscript: CZ and XL. All authors contributed to the article and approved the submitted version.
