# Supplementary material for: Single-cell and bulk RNA sequencing analysis of B cell marker genes in TNBC TME landscape and immunotherapy
Source: Front Immunol. 2023 Dec 4;14:1245514. doi: 10.3389/fimmu.2023.1245514 (PMC10725955; doi:10.3389/fimmu.2023.1245514)
Supplement: Supplementary file 2 [file Table_2.docx]

| **Clinical characteristics** | | **Number and percentage of TNBC patients** | |
| --- | --- | --- | --- |
|  |  | TCGA (%) | GSE58812 (%) |
| **Total** |  | 160 | 107 |
| **Age (years)** | ≤60 | 110 (68.75%) | 84 (78.50%) |
|  | >60 | 50 (31.25%) | 23 (14.37%) |
| **Stage-AJCC** | I | 29 (18.12%) | 18 (11.25%) |
|  | II | 101 (63.12%) | 71 (44.37%) |
|  | III | 25 (15.62%) | 15 (9.37%) |
|  | IV | 5 (3.12%) | 3 (2.80%) |

Table S2. Clinical characteristics of the training group TCGA and the testing group GSE58812.
